# Lysimachiae Herba Inhibits Inflammatory Reactions and Improves Lipopolysaccharide/D-Galactosamine-Induced Hepatic Injury

**DOI:** 10.3390/antiox10091387

**Published:** 2021-08-30

**Authors:** Yun Hee Jeong, Tae In Kim, You-Chang Oh, Jin Yeul Ma

**Affiliations:** Korean Medicine (KM)-Application Center, Korea Institute of Oriental Medicine, 70 Cheomdanro, Dong-gu, Daegu 41062, Korea; runxi0333@kiom.re.kr (Y.H.J.); tikim@kiom.re.kr (T.I.K.)

**Keywords:** Lysimachiae Herba, anti-inflammatory, antioxidant, hepatoprotective, lipopolysaccharide, D-galactosamine

## Abstract

This study aimed to determine the anti-inflammatory and hepatoprotective effects of Lysimachiae Herba ethanolic extract (LHE) in lipopolysaccharide (LPS)-stimulated macrophages and in a LPS/D-galactosamine (GalN)-induced acute hepatitis mouse model. Then, the production of inflammatory mediators and the activation of related pathways in macrophages were explored. Finally, we assessed the serum aminotransferase levels and the expression of inflammatory/antioxidant molecules in liver tissues in mice. Results revealed that LHE treatment significantly inhibited the production of inflammatory mediators in LPS-stimulated RAW 264.7 macrophages. Molecular data showed that LHE remarkably increased the activities of the antioxidant pathway and inhibited the phosphorylation of mitogen-activated protein kinase as well as the transcriptional activity of nuclear factor-κB induced by LPS. Furthermore, it prevented acute liver damage caused by LPS/D-GalN-induced hepatitis by inhibiting aminotransferase levels and histopathological changes in mice. Moreover, treatment with LHE significantly inhibited the activation of inflammatory pathways and increased the expression of antioxidant molecules including heme oxygenase-1/Nuclear factor erythroid 2-related factor 2. In conclusion, LHE has potent anti-inflammatory and hepatoprotective effects in LPS-stimulated macrophages and the LPS/D-GalN-induced acute hepatitis mouse model. Thus, it can be a treatment option for inflammation, hepatitis, and liver injury.

## 1. Introduction

Lysimachiae Herba (LH) is a dried whole part of *Lysimachia christinae*, known in Korea as “Geumjeoncho”, and is commonly found in East Asia. This herb has been commonly used as a traditional herbal medicine for the treatment of inflammatory diseases, viral hepatitis, cholecystitis, urinary stones, and jaundice [1,2,3]. LH contains flavonoids and phenols as major components, and these compounds have been studied for their anti-cholecystitis efficacy and cholagogic activity [4]. Many previous studies have investigated the efficacy of LH on liver-related diseases, but scientific studies of its anti-inflammatory properties and precise cellular molecular mechanisms are lacking, and the effect of LH on lipopolysaccharide (LPS)/D-galactosamine (GalN)-induced acute hepatitis has not been studied.

Fulminant hepatitis is a severe liver disease pathologically by extensive hepatocellular apoptosis and hemorrhagic necrosis leading to multiple organ failures [5,6]. Fulminant hepatic injury is caused by a variety of factors that can cause liver failure, including alcohol, chemicals, oxidizing agents, and hepatitis virus [7]. LPS and D-GalN are the most common agents that cause fulminant hepatic injury [8,9]. LPS is a component of the gut Gram-negative bacteria [10], and plays an important role in the initiation stage of endotoxic damage and activates inflammatory cytokines, causing liver tissue damage. D-GalN causes hepatocyte necrosis upon acute exposure, and cirrhosis and cell tumor during chronic exposure [11]. Additionally, D-GalN is able to sensitize toxic effects of the liver toward endotoxins such as LPS and results in fulminant hepatic failure within a few hours [12]. LPS/D-GalN exposure results in liver damage due to overgrowth of intestinal bacteria, disruption of intestinal barrier function, and an increase in permeability to endotoxin and bacteria [13]. Therefore, hepatic injury induced by LPS in combination with D-GalN is well-accepted animal model which is significantly similar to acute hepatic failure in the clinical setting [7] and is widely used to study the underlying mechanism of fulminant hepatitis and its potential therapeutic drugs [14,15]. Previous studies reported that LPS/D-GalN induced secretion of inflammatory cytokines, such as tumor necrosis factor (TNF)-α, interleukin (IL)-6, and IL-1β in mice model of hepatic liver injury [16,17], which promote liver cell necrosis, hepatic failure [18,19] and reduce antioxidant enzyme activity [20]. Therefore, reducing the inflammatory cytokines and activating the antioxidant enzymes may be key to the prevention and treatment of fulminant hepatitis.

The production of cytokines is closely involved in the activation of mitogen-activated protein kinase (MAPK) and nuclear factor (NF)-κB signaling pathway [21]. When acute liver injury is induced with LPS/D-GalN, LPS binds to the Toll-like receptor of Kupffer cells and promotes the transcription and production of inflammatory cytokines such as TNF-α, IL-6, and IL-1β [22,23]. Thus, NF-κB and MAPK signaling pathway are critical contributors to the mechanism of the LPS/D-GalN-induced fulminant hepatitis. Nuclear factor E2-related factor 2 (Nrf-2) is a major transcription factor that regulates cellular defense mechanism against oxidative stress and inflammation by regulating the level of several endogenous cytoprotective enzyme, including heme oxygenase (HO)-1. Previous studies reported that the protective effect of the Nrf-2 pathway in acute liver inflammation and liver fibrosis [24,25,26]. Another study has shown that HO-1 has anti-inflammatory activity against acute and chronic inflammatory diseases, including experimental colitis, bronchitis, and hepatitis [27,28]. Therefore, Nrf-2/HO-1 signaling pathway plays a positive role in the pathogenesis of inflammatory diseases including fulminant hepatitis induced by LPS/D-GalN. Based on these previous findings, we also hypothesized that the hepatoprotective effect of LHE may be closely associated with strong anti-inflammatory activity. Therefore, in this study, we not only investigated the hepatoprotective effect of LHE on LPS/D-GalN-induced acute hepatitis mice models in vivo, but also investigated whether LHE has an inhibitory effect on inflammatory responses in RAW 264.7 macrophages stimulated with LPS in vitro levels. We also explored chemical components of LHE by high-performance liquid chromatography (HPLC) analysis.

## 2. Materials and Methods

### 2.1. Materials and Reagents

Roswell park memorial institute (RPMI) 1640 medium, fetal bovine serum (FBS), and antibiotics were obtained from Hyclone (Logan, UT, USA). LPS, dexamethasone (Dex), bovine serum albumin (BSA), and D-GalN were purchased from Sigma-Aldrich (St. Louis, MO, USA). Enzyme-linked immunosorbent assay (ELISA) antibody sets were obtained from eBioscience (San Diego, CA, USA). Cell culture dish and well plates were purchased from SPL Life Sciences (Pocheon, Korea). The cell counting kit (CCK) was obtained from Dojindo Molecular Technologies, Inc. (Kumamoto, Japan). Bradford reagent was purchased from Bio-Rad (Hercules, CA, USA). Nitrocellulose (NC) membrane was obtained from Millipore (Bedford, MA, USA). Various primary and horseradish peroxidase (HRP)-conjugated secondary antibodies for protein analysis were purchased from Cell Signaling Technology, Inc. (Boston, MA, USA). RNA extraction reagent and DNA synthesizing kit were obtained from iNtRON Biotech (Daejeon, Korea). Oligonucleotide primers for tumor necrosis factor (TNF)-α, interleukin (IL)-6, IL-1β, inducible nitric oxide synthase (iNOS), cyclooxygenase (COX)-2, HO-1, and β-actin were synthesized from Bioneer (Daejeon, Korea). The quantitative polymerase chain reaction (qPCR) reaction reagent kit was purchased from Bioneer. Protocatechuic acid, catechin, quercitrin, quercetin, and kaempferol were purchased from Sigma-Aldrich. HPLC-grade acetonitrile was provided by Merck KFaA (Darmstadt, Germany). ACS reagent-grade formic acid was obtained from Sigma-Aldrich. Water analyzed via HPLC was prepared using the Puris-Evo RO water system (Mirae ST Co., Ltd., Anyang-si, Korea).

### 2.2. Preparation of LHE

LH was purchased as a dried herb from Yeongcheonhyundai Herbal Market (Yeongcheon, Korea) and was identified by Prof. KiHwan Bae (College of Pharmacy, Chungnam National University, Korea). All voucher specimens were deposited in an herbal bank at the KM-Application Center, Korea Institute of Oriental Medicine (voucher number: E207). The dried herb (30.0 g) was extracted with 390 mL of 70% ethanol in a 40 °C shaking incubator (100 rpm) for 24 h. In some previous studies using plant extracts, 70% ethanol was used as the extraction solvent, and we set the extraction conditions for LH with reference to the studies [29,30]. The extract solution was filtered using a 150 mm filter paper (Whatman, Piscataway, NJ, USA) and was concentrated to 100 mL using a rotary vacuum evaporator (Buchi, Tokyo, Japan). Samples were then freeze-dried and stored in desiccators at −20 °C before use. The sample yield was 12.2627%, and 3.6788 g of extract was obtained.

### 2.3. Cell Culture and Drug Treatment

Murine macrophage RAW 264.7 cells were obtained from Korea Cell Line Bank (Seoul, Korea) and were cultured in complete RPMI 1640 medium (10% FBS and 1% antibiotics (*v*/*v*)). The cells were then incubated in humidified 5% CO_2_ atmosphere at 37 °C. To stimulate the cells, LPS (200 ng/mL) was added in the presence or absence of LHE (10, 100, or 200 μg/mL) at the indicated periods.

### 2.4. Cell Viability Assay

LHE-induced cytotoxicity was analyzed using CCK reagent. The macrophages were seeded in 96-well culture plates at a density of 5 × 10^4^ cells/well in 100 μL medium. After 18 h of incubation, LHE was added to the cells and was incubated for 24 h at 37 °C with 5% CO_2_. Then, 10 μL of CCK solutions was applied to each well, and the cells were incubated for another 1 h. Then, the optical density was read at 450 nm using ELISA reader (infinite M200, TECAN, Mannedorf, Switzerland).

### 2.5. Analysis of NO Production

NO production was analyzed by measuring nitrite levels in the supernatants of cultured macrophage cells. Macrophage cells (5 × 10^4^ cells/well in 100 μL medium) were plated, incubated with LHE, and stimulated with LPS for 24 h. The supernatant was mixed with the same volume of Griess reagent (1% sulfanilamide, 0.1% naphthylethylenediamine dihydrochloride, and 2.5% phosphoric acid) and was incubated at room temperature (RT) for 5 min. The absorbance at 570 nm was read. The concentration of nitrite was calculated with sodium nitrite as the standard.

### 2.6. Inflammatory Cytokine Production

To determine the effects of LHE, the production of pro-inflammatory cytokines, such as TNF-α, IL-6, and IL-1β, was assessed using ELISA. That is, 2.5 × 10^5^ cells/well in 500 μL medium were seeded on 24 well plates and were incubated overnight. The cells were pretreated with three concentrations of LHE for 1 h and further challenged with LPS for an additional 24 h at 37 °C with 5% CO_2_. The cytokine levels in the supernatants were measured using ELISA antibody sets according to the manufacturer’s instructions.

### 2.7. Preparation of Whole Cell, Cytosolic, Nuclear, and Mice Liver Tissue Extracts

To obtain whole cell and liver tissue lysates, cell pellets or mouse liver tissue samples were resuspended in radioimmunoprecipitation assay lysis buffer (Millipore) containing protease and phosphatase inhibitors. Cytosol and nuclear fractions were isolated using NE-PER nuclear and cytoplasmic extraction reagents (Thermo Scientific, Rockford, IL, USA), according to the manufacturer’s instruction. The fractions were stored at −80 °C before use.

### 2.8. Western Blotting for Protein Analysis

Western blotting was performed to evaluate the effects of LHE on the expression of various inflammatory response-related proteins or inflammatory pathway proteins in the whole cell, cytosol, nucleus, or mice liver tissues. For cell protein analysis, the cells were pretreated with LHE and stimulated with LPS at indicated periods. After incubation, the cells were collected via scrapping and were washed twice with ice-cold phosphate buffered saline (PBS). For liver protein analysis, mouse liver tissue samples were collected and gently rinsed twice with PBS. The total proteins were determined using the Bradford’s method. Equal amounts of proteins were subjected to sodium dodecyl sulfate–polyacrylamide gel electrophoresis after transferring them into an NC membrane with a glycine transfer buffer (192 mM glycine, 25 mM Tris-HCl [pH 8.8], and 20% MeOH [*v*/*v*]). Then, after blocking the nonspecific site with 3% BSA, the membrane was then incubated with specific primary antibody at 4 °C overnight. Next, it was subsequently incubated with HRP-conjugated secondary antibodies. The specific proteins were detected using SuperSignal West Femto Chemiluminescent Substrate (Thermo Scientific). Protein levels were quantified using a ChemiDoc^TM^ Touch Imaging System (Bio-Rad). Table 1 shows the information about various primary and secondary antibodies.

### 2.9. RNA Extraction, DNA Synthesis, and qPCR

Total cellular RNA was isolated using the easy-BLUE™ RNA extraction kit according to the manufacturer’s instruction. The total RNA (1 μg) was reversed transcribed into cDNA using RevoScript™ RT PreMix. Table 2 shows the oligonucleotide primer sequences for qPCR. The reactions were setup in triplicates with a total volume of 20 μL: final concentration of 0.3 μM for each primer, 10 μL of AccuPower^®^ 2X Greenstar qPCR Master Mix, and 2 μL of template DNA. The following qPCR conditions were applied for TNF-α, IL-6, IL-1β, iNOS, COX-2, HO-1, and β-actin: 40 cycles at 94 °C for 15 s and 60 °C for 1 min [31]. Amplification and analyses were performed using QuantStudio 6 Flex Real-time PCR System (Thermo Scientific). Samples were compared using the relative CT method. The fold increase or decrease in gene expression was determined relative to a blank control after normalization to β-actin gene using 2^−ΔΔC^T [31].

### 2.10. Animals Used for the Analysis of LPS/D-GalN-Induced Acute Hepatitis

Six-week-old male imprinting control region (ICR) mice (30 ± 3 g) were purchased from Samtako BioKorea (Osan, Korea). All animals were stored in a room with controlled temperature under a 12 h light/12 h dark cycle, with food and water provided ad libitum. Mice were fed gamma irradiation-sterilized LabDiet 5053 (Orient, Seongnam, Korea), which included protein 20%, fat (ether extract) 4.5%, fat (acid hydrolysis) 5.4% crude fiber 4.7%, ash 6%, calcium 0.8%, and phosphorus 0.62%. All mice were acclimatized for at least 7 days prior to the experiments. All experimental procedures were performed in accordance with the guidelines for the Animal Care and Use Committee of Korea Institute of Oriental Medicine (reference number: #D-17-020).

### 2.11. Protocol for the LPS/D-GalN-Induced Hepatitis Mouse Model

Mice were randomly divided into four groups, which are as follows: normal group (vehicle, 0.5% carboxymethyl cellulose [CMC]), LPS/D-GalN group (50 μg LPS and 1 g D-GalN/kg), LHE along group (300 mg/kg LHE), and test group (LPS/D-GalN + 100, 200, or 300 mg/kg of LHE). The dose of LHE administered to mice was set with reference to a previous study [32], and a group administered with 300 mg/kg of LHE alone was analyzed to exclude potential toxicity caused by LHE administration. Briefly, three concentrations of LHE were administered orally to mice for a total of 6 days once a day (diluted in a volume of 10 mL/kg of 0.5% CMC). During this period, the normal and LPS/D-GalN groups received the same amount of vehicle. One hour after the last injection, LPS/D-GalN was administered intraperitoneally in each mouse. Next, the mice were sacrificed after 6 h. Mouse liver tissues and serum samples were collected and used for hematoxylin and eosin (H&E) staining, Western blotting, ELISA, and aminotransferase analysis.

### 2.12. Biochemical Analysis for the Evaluation of Serum Alanine Aminotransferase, Aspartate Aminotransferase, Alkaline Phosphatase, and Liver Tissue Protein Levels in Mice

All mice were euthanized 6 h after the administration of LPS/D-GalN. Then, liver tissue and blood samples were collected for biochemical analysis. Mice serum was separated from the blood via centrifugation at 2000× *g* for 15 min. The serum alanine aminotransferase (ALT), aspartate aminotransferase (AST), and alkaline phosphatase (ALP) levels were assessed using an Erba XL-200 automated clinical chemistry analyzer (Mannheim, Germany). To analyze the expression of different inflammatory proteins, the liver tissue was homogenized and dissolved in radioimmunoprecipitation assay buffer. All protein analysis results were normalized using the β-actin of each sample to make the total protein amount constant.

### 2.13. Histopathological Examination

Histological analysis of mice liver was performed to evaluate the inhibitory efficacy of LHE on the histological changes in liver tissues caused by LPS/D-GalN. After the mice were euthanized, the liver tissues were separated, fixed in 10% formaldehyde for 10 days, embedded in paraffin wax, and cut into sections with a thickness of 5 μM. Paraffin-embedded sections were stained with H&E and were subjected to pathological analysis under a microscope. Sections were assessed for liver injury with Axioskop 40 (Oberkochen, Germany) and were photographed at 400× magnification.

### 2.14. HPLC Instruments

HPLC analysis was conducted using the Dionex Ultimate 3000 system set up with a column oven, auto sampler, binary pump, and diode assay UV/VIS detector (Dionex Corp., Sunnyvale, CA, USA). Data acquisition and processing were performed using Chromeleon 7 (Thermo Fisher, Counteaboeuf, France).

### 2.15. Preparation of Plant Material Sample and Standard

In total, 100 mg of LHE was precisely weighted and dissolved in 1 mL of HPLC-grade methanol using an ultrasonicator (JAC Ultrasonic JAC-3010) for 30 min. The LHE solution sample at a concentration of 100 mg/mL was filtered using a 0.2 PETE membrane syringe filter. After filtration, 10 μL of filtrate was injected for HPLC analysis. To confirm each standard retention time using the established method, protocatechuic acid, catechin, quercitrin, quercetin, and kaempferol standard solutions were prepared at 1.0 mg/mL with methanol. Then, 10 μL of standard solution filtrate was analyzed using the HPLC system. After analysis, the retention time of LHE and each standard compound were compared. Moreover, to confirm the content of each compound in LHE, we prepared a standard curve for several compounds, which were diluted with methanol at different concentrations.

### 2.16. HPLC Analysis

To identify the content of five major compounds (protocatechuic acid, catechin, quercitrin, quercetin, and kaempferol) in LHE, we conducted an HPLC analysis, which was operated with C18 column with a C18 guard cartridge (4.0 × 3.0 mm). The mobile phase was set up with A, 1.0% formic acid and B, acetonitrile was eluted at a flow rate of 1 mL/min. The LHE and standard compound solution were analyzed using the established gradient mobile phase method. The HPLC chromatogram of LHE and the standard compound was detected under HPLC conditions, which included UV detection at 270 nm, column oven temperature of 40 °C, and injection volume of 10 μL (Table 3). Calibration curves, assessed using the standard solution and the limits of detection and quantification (LOQ) under chromatographic conditions, were determined by injecting a series of standard solutions. The result was processed using Chromeleon 7 (Thermo Fisher), and the solutions were administered three times under the same condition.

### 2.17. Statistical Analysis

Data were expressed as means ± standard error of the mean (SEM) for all experiments, and all quantitative data were representative of at least three independent experiments. Statistical significance was determined via one-way analysis of variance, followed by the Dunnett’s test after comparing each treatment group and LPS or LPS/D-GalN. * *p* values of <0.05, ** < 0.01, and *** < 0.001 (vs. LPS or LPS/D-GalN) were considered statistically significant.

## 3. Results

### 3.1. Effect of LHE on LPS-Induced NO Production and Cytotoxicity

**Figure 1:**
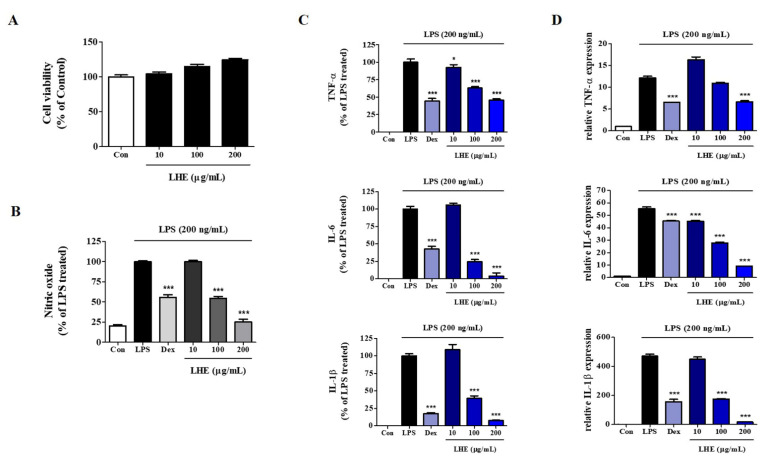
Effects of LHE on (**A**) cell viability, production of (**B**) NO and (**C**) inflammatory cytokines, and (**D**) expression of their mRNA in LPS-stimulated RAW 264.7 macrophages. Control cells were incubated with vehicle alone. Data were presented as mean ± SEM of determinations from three independent experiments. * *p* < 0.05 and *** *p* < 0.001 were calculated by comparing LPS stimulation values.

We explored the influence of LHE on the proliferation and viability of RAW 264.7 cells using the CCK assay. As shown in Figure 1A, LHE treatment did not show cytotoxicity up to 200 μg/mL. Thus, we used less than 200 μg/mL of LHE in subsequent experiments. Next, we examined the inhibitory effect of LHE on the production of NO from LPS-stimulated RAW 264.7 cells. The expression of NO in culture media was quantified using Griess reagent assays. LPS alone significantly increased nitrite levels in the cells compared with controls. However, pretreatment of cells with LHE significantly inhibited the production of LPS-induced nitrite in a concentration-dependent manner (Figure 1B).

### 3.2. Effects of LHE on LPS-Induced Pro-Inflammatory Cytokine Production

LPS-stimulated macrophages secrete pro-inflammatory cytokines, such as TNF-α, IL-6, and IL-1β, which are essential factors for inflammatory disease [33]. Thus, we determined the effects of LHE on the production of pro-inflammatory cytokines and their mRNAs in RAW 264.7 cells treated with LPS. Cells were pretreated with LHE for 1 h and stimulated for 24 h via LPS. Next, the levels of cytokines were analyzed using ELISA and qPCR. As shown in Figure 1C, LHE strongly decreased LPS-induced TNF-α, IL-6, and IL-1β cytokine secretion in a concentration-dependent manner. Moreover, the inhibitory rates were 54%, 96%, and 92% at a concentration of 200 μg/mL, respectively. Similarly, based on the qPCR results, LHE significantly decreased the mRNA expression of TNF-α, IL-6, and IL-1β via LHE treatment in a concentration-dependent manner (Figure 1D).

### 3.3. Effects of LHE on LPS-Induced iNOS and COX-2 Expression

**Figure 2:**
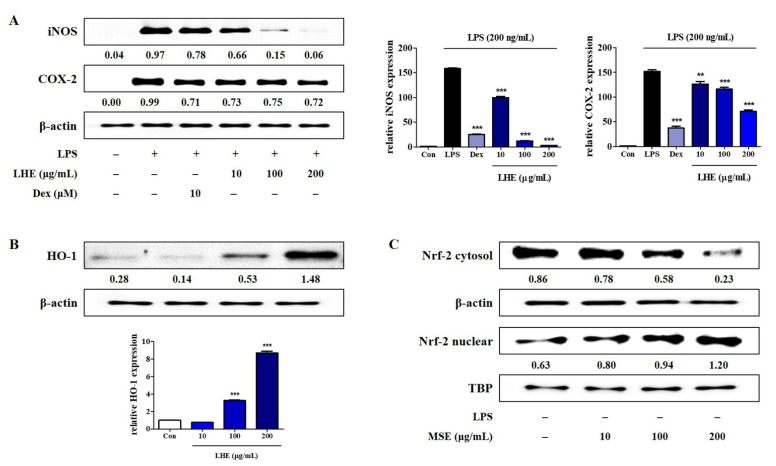
Effects of LHE on (**A**) iNOS and COX-2 expression, (**B**) HO-1 induction, and (**C**) nuclear translocation of Nrf-2 in RAW 264.7 macrophages. (**A**) Cells were incubated with LHE for 1 h and then stimulated with LPS for 24 h (protein) or 12 h (mRNA). Cells were incubated with LHE alone for (**B**) 6 h or (**C**) 3 h. The quantified number and histograms showed the expression levels of protein and mRNA relative to those of β-actin or TBP. ** *p* < 0.01 and *** *p* < 0.001 were calculated by comparing LPS stimulation values.

NO and PGE_2_ expressions are involved in the regulation of iNOS and COX-2 levels in RAW 264.7 cells, respectively. Thus, we assessed the inhibitory effects of LHE on the protein and mRNA expression of iNOS and COX-2 via Western blotting and qPCR. As shown in Figure 2A, compared with LPS treatment, pretreatment with LHE significantly reduced the expression of protein and mRNA genes of iNOS and COX-2 in a concentration-dependent manner.

### 3.4. Effects of LHE on HO-1 Expression and Nrf-2 Nuclear Translocation

HO-1 is an important enzyme, and it plays an essential role due to its anti-inflammatory effects in RAW 264.7 cells [34]. Moreover, the induction of HO-1 is mediated by Nrf-2 activation via migration into nucleus. Thus, the influence of LHE on HO-1 expression and nuclear Nrf-2 accumulation was examined using Western blotting and qPCR. As shown in Figure 2B,C, LHE treatment significantly enhanced the expression of HO-1 and nuclear Nrf-2 at a concentration of 200 μg/mL.

### 3.5. Effects of LHE on LPS-Induced Activation of the MAPK Signaling Pathway

**Figure 3:**
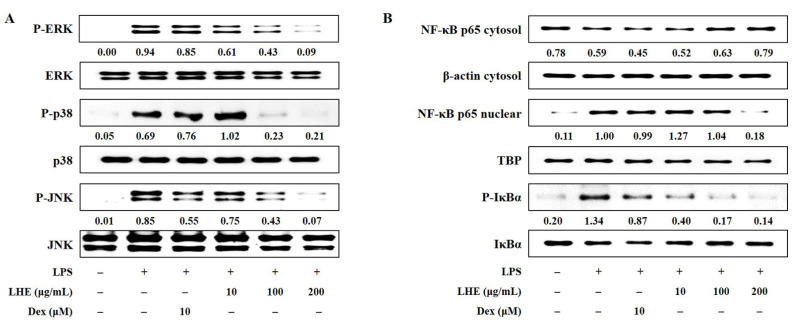
Effects of LHE on (**A**) the phosphorylation of MAPK and (**B**) the activation of NF-κB in LPS-stimulated RAW 264.7 macrophages. Cells were stimulated with LPS for (**A**) 30 min or (**B**) 1 h. The quantified number showed the expression levels of protein relative to those of total-type protein, β-actin, or TBP.

Three families of MAPK including extracellular signal-regulated kinase (ERK), p38, and c-Jun-NH_2_-terminal kinase (JNK) play an important role in LPS-induced inflammatory responses in macrophages. Thus, the effects of LHE on LPS-stimulated phosphorylation of MAPK were assessed via Western blotting. Our data showed that the activation of MAPK increased after exposure to LPS at a dose of 200 ng/mL. However, LHE treatment was exclusive, and it significantly inhibited the phosphorylation of ERK, p38, and JNK MAPK in a dose-dependent manner (Figure 3A).

### 3.6. Effects of LHE on the Transcriptional Activity of NF-κB

NF-κB is a transcription factor that has a key role in inflammatory responses. To investigate whether the anti-inflammatory activity of LHE against the NF-κB signaling pathway in RAW 264.7 cells, we evaluated whether LHE affects LPS-induced NF-κB activation and inhibitor of NF-κB alpha (IκBα) phosphorylation. Western blotting revealed that LHE pretreatment significantly inhibited p65 nuclear localization (Figure 3B). That is, it has a higher inhibitory effect than Dex when used as a positive control at a concentration of 200 μg/mL. Consistent with these results, LHE can remarkably attenuate LPS-induced degradation and the phosphorylation of IκBα in the cytoplasm.

### 3.7. Effects of LHE on LPS/D-GalN-Induced Liver Injury in Mice

**Figure 4:**
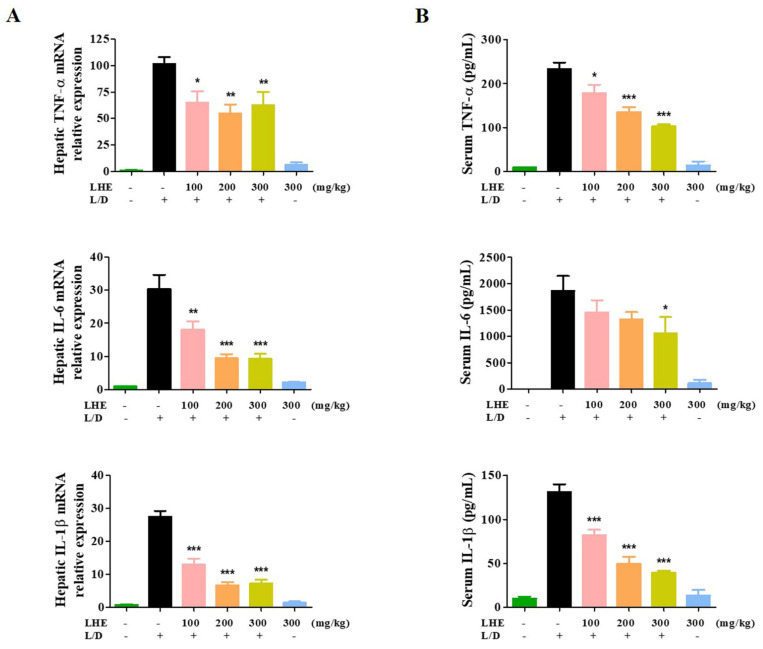
Effects of LHE on (**A**) the expression of hepatic cytokine mRNA and (**B**) serum cytokine levels in LPS/D-GalN-induced hepatitis mouse model. Mice were pretreated with LHE or vehicle once per day for 6 days. Then, LPS/D-GalN was injected 1 h after the last administration. After 6 h, blood samples were collected from the abdominal vena cava puncture, and serum was prepared via centrifugation. (**A**) The mRNA levels of hepatic cytokine were analyzed via qPCR. (**B**) Serum cytokine levels were determined via ELISA. Data were expressed as mean ± SEM (*n* = 9). * *p* values of < 0.05, ** < 0.01, and *** < 0.001 (vs. LPS/D-GalN) were considered statistically significant.

**Figure 5:**
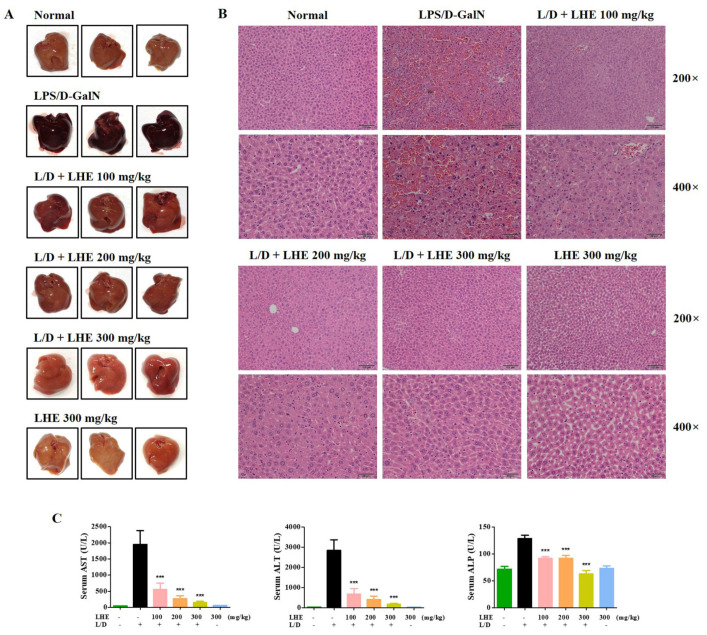
Effects of LHE on (**A**) mouse liver injury, (**B**) histopathological changes of mouse liver, and (**C**) serum aminotransferase levels in LPS/D-GalN-induced hepatitis mouse model. Mice were pretreated with LHE or vehicle once per day for 6 days. Then, LPS/D-GalN was injected 1 h after the last administration. After 6 h, mice were sacrificed, and liver tissue and blood samples were collected. Mouse serum was prepared via centrifugation. (**A**) Images of hepatitis lesions in mice. (**B**) H&E staining of mice liver. Scale bars = 100 μm (200×) or 50 μm (400×). (**C**) Serum aminotransferase levels were analyzed using an automated clinical chemistry analyzer. Data were expressed as means ± SEM (*n* = 9). *** *p* values of < 0.001 (vs. LPS/D-GalN) were considered statistically significant.

We explored the hepatoprotective effects of LHE via LPS/D-GalN-induced hepatic damage in vivo. The ICR mice received different doses of LHE, including 100, 200, and 300 mg/kg with LPS/D-GalN, or 300 mg/kg of LHE alone. First, we evaluated several pro-inflammatory cytokines in liver tissues and serum via qPCR and ELISA. As shown in Figure 4, LHE could effectively inhibit the expression of TNF-α, IL-6, and IL-1β cytokine and their mRNA levels in liver tissues and serum. The level of cytokine in the 300 mg/kg LHE/no injected LPS/D-GaIN group was close to that of the normal group. As shown in Figure 5A, compared with the normal group, the LPS/D-GalN group had severe liver damage. However, the livers of the LHE group were morphologically comparable with those of the normal group in a dose-dependent manner. Moreover, the 300 mg/kg LHE alone group was morphologically similar to the normal group. Hence, LHE at a dose of up to 300 mg/kg could prevent hepatotoxicity, and it had protective effects against acute hepatitis. Moreover, H&E staining of liver tissues revealed that the normal and 300 mg/kg LHE alone group had no pathological abnormalities (Figure 5B). However, the LPS/D-GalN group resulted in severe histopathological changes in the liver, such as extensive hemorrhage, necrosis, and neutrophil infiltration. The LHE group enhanced LPS/D-GaIN-induced liver injury in a dose-dependent manner (Figure 5B).

### 3.8. Effects of LHE on LPS/D-GalN-Induced Serum ALT, AST, and ALP Levels

Since LHE has strong anti-inflammatory effects against LPS-induced inflammation in macrophages, we assessed whether LHE could effectively prevent LPS/D-GalN–induced severe hepatitis in mice. ALT, AST, and ALP are important markers in evaluating hepatic injury. As depicted in Figure 5C, the serum ALT, AST, and ALP levels significantly increased in the LPS/D-GalN group compared with the normal group. However, the administration of LHE can effectively inhibit the expression of ALT and AST in a dose-dependent manner (Figure 5C). At the ALP level, the inhibition rates were similar at 100 and 200 mg/kg, and a more remarkable effect was detected at 300 mg/kg (Figure 5C).

### 3.9. Effects of LHE on Hepatic Activities of HO-1 and Nrf-2

**Figure 6:**
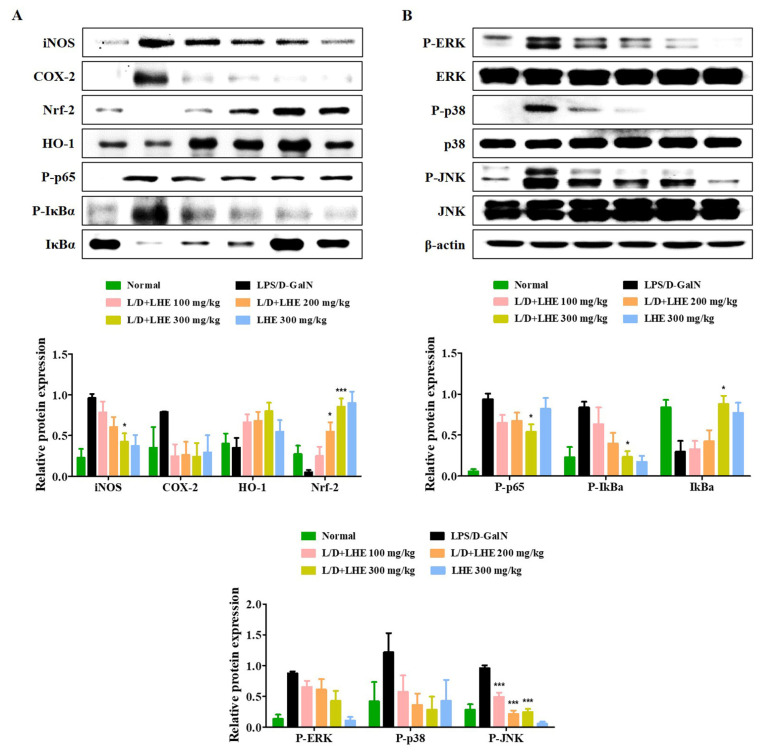
Effects of LHE on (**A**) the expression of iNOS/COX-2, induction of HO-1/Nrf-2, activation of NF-κB, and (**B**) phosphorylation of MAPK in LPS/D-GalN-induced hepatitis mouse model. Mice were pretreated with LHE or vehicle once per day for 6 days. Then, LPS/D-GalN was injected 1 h after the last administration. After 6 h, mice were sacrificed, and liver tissue samples were collected. The expression of inflammatory synthetic enzyme, antioxidant molecules, and inflammatory pathways were determined via Western blot analysis. The histograms showed the expression levels of protein relative to those of β-actin. * *p* values of < 0.05, and *** < 0.001 (vs. LPS/D-GalN) were statistically significant.

The Nrf-2/HO-1 pathway is an important defense molecular mechanism in oxidative stress and inflammation to inhibit hepatitis. Our results showed that pretreatment with LHE has anti-inflammatory effects via the activation of Nrf-2/HO-1 in RAW 264.7 macrophages. Therefore, we investigated whether LHE has effects on Nrf-2 and HO-1 expression in the LPS/D-GalN-induced mouse model. As shown in Figure 6A, the level of Nrf-2 and HO-1 significantly decreased in the LPS/D-GalN group compared with the normal group. In contrast, the administration of LHE could significantly increase the expression of Nrf-2 and HO-1 in a dose-dependent manner, which has important protective effects against LPS/D-GalN-induced liver failure (Figure 6A). In addition, LHE is effective in inhibiting the expression of hepatic iNOS and COX-2 proteins in mice (Figure 6A).

### 3.10. Effects of LHE on the Hepatic Activities of the NF-κB and MAPK Signaling Pathways

Our results in vitro showed that pretreatment with LHE had anti-inflammatory effects by inhibiting the NF-κB and MAPK signaling pathways. Therefore, whether hepatitis was inhibited via the regulation of NF-κB and MAPK in the LPS/D-GalN-induced mouse model was further assessed via Western blotting. As shown in Figure 6A,B, the activation of NF-κB and MAPK was significantly induced in the LPS/D-GalN group compared with the normal group. However, the phosphorylation of NF-κB p65 and three MAPK as well as the degradation of IκBα were reduced in a dose-dependent manner in the LHE group.

### 3.11. Content of Major Compounds in LHE

**Figure 7:**
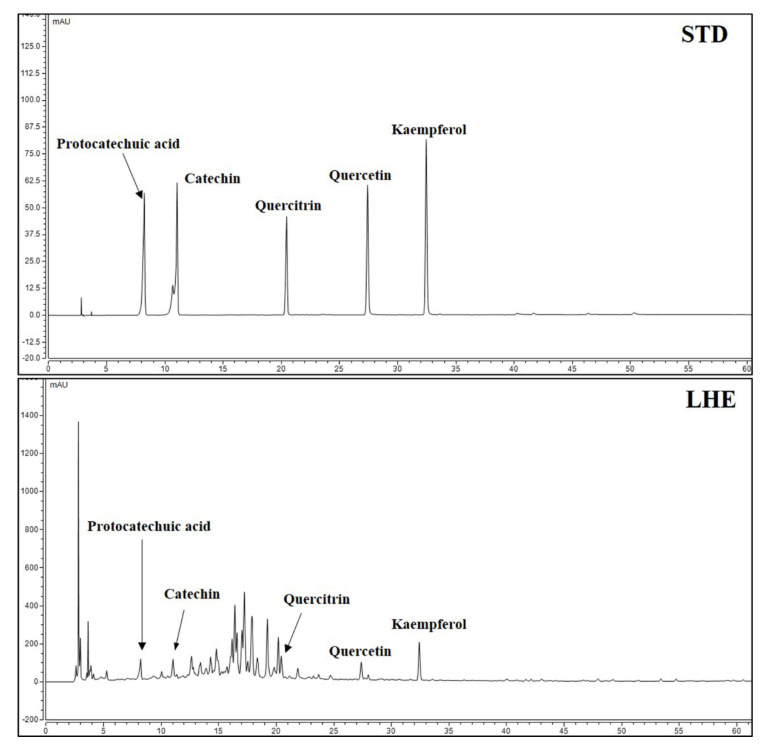
HPLC chromatograms of standard solution and LHE at 270 nm.

Five major compounds and LHE were analyzed using the established HPLC system. Thus, protocatechuic acid, catechin, quercitrin, quercetin, and kaempferol were successively detected at 8.250, 11.067, 20.453, 27.423, and 32.470 min, respectively (Figure 7). A calibration curve of the major compounds (protocatechuic acid, catechin, quercitrin, quercetin, and kaempferol) was prepared to determine the amount of each component within LHE. The calibration curve linearity of the five major compounds was good at the tested concentration range (Table 4). Each compound area mean value in LHE was calculated using the calibration curve equation, which was prepared at the tested concentration range. Hence, the contents were 0.03% protocatechuic acid, 0.30% catechin, 0.04% quercitrin, 0.03% quercetin, and 0.04% kaempferol (Table 5).

## 4. Discussion

The liver is a vital organ and is easily damaged by different factors, thereby leading to liver failure. These factors include alcohol use and exposure to chemical substances, oxidative products, and hepatitis virus [7]. Hepatitis is characterized by inflammatory conditions in the liver, and it can be self-limiting or can lead to liver fibrosis, cirrhosis, and cancer. The major causes of hepatitis are viruses, alcohol use, exposure to toxins, intake of certain drugs, other infections, autoimmune diseases, and non-alcoholic steatohepatitis [35]. The role of gut-derived LPS in the pathogenesis of hepatic injury has been widely revealed [36]. LPS is the primary endogenous endotoxin of Gram-negative bacteria in the gut that induces a strong inflammatory response, which can lead to liver tissue injury [37,38]. D-GaIN is a specific hepatotoxicant that used to increase the sensitivity to the lethal effects of endotoxin such as LPS [12]. Additionally, LPS/D-GalN exposure results in fulminant hepatitis due to overgrowth of bacteria in the gut, disruption of intestinal barrier function, and an increase in permeability to endotoxin and bacteria [13]. Thus, LPS/D-GalN-induced acute liver injury is a well-established experimental model to investigate the underlying mechanisms for fulminant hepatitis and screen potential therapeutic drugs [7]. Therefore, to investigate the therapeutic effects of LHE on inflammatory symptoms in vivo, we determined the hepatoprotective effects using LPS/D-GalN-induced acute hepatitis mice model.

Excessive production of inflammatory cytokines, such as TNF-α, IL-6, and IL-1β are closely associated with LPS/D-GalN-induced liver injury. These inflammatory cytokines have the ability to induce hepatocyte apoptosis and necrosis [39]. Thus, we examined the inhibitory effects of LHE on inflammatory cytokine levels. The ICR mice received LHE at different doses including 100, 200, and 300 mg/kg with LPS/D-GalN injection, or LHE at a dose of 300 mg/kg alone. We found that injection of LPS/D-GalN dramatically induced the expression of inflammatory cytokines. However, administration of LHE significantly inhibited the production of inflammatory cytokines and their mRNA genes such as TNF-α, IL-6, and IL-1β in a dose-dependent manner. Additionally, ALT and AST are known as representative indicators of hepatocellular damage, indicating the degree of liver injury [40]. Thus, we determined the concentrations of ALT, AST, and ALP in the mouse serum of each group. Our results indicated that LHE efficiently repressed the levels of ALT, AST, and ALP in serum compared with the LPS/D-GalN group. In addition, LHE significantly decreased LPS/D-GalN-triggered liver functional damage in a dose-dependent manner. Moreover, the 300 mg/kg LHE/no injected LPS/D-GaIN group was morphologically comparable with the normal group. Hence, LHE of a dose up to 300 mg/kg could not only prevent hepatotoxicity but also protect the liver. These experimental data revealed that LHE exerts protective effects against LPS/D-GalN-induced hepatic damage by inhibiting inflammatory response.

The signal cascades that control pro-inflammatory cytokine expression is deeply associated with the MAPK-mediated signaling pathway [21]. The MAPK signaling pathway has been known to play a critical role in regulating inflammatory factors [41]. Several studies have reported that the phosphorylation of MAPK by LPS-stimulation is involved in the upregulation of pro-inflammatory factors via the activation of NF-κB [42,43]. NF-κB is a transcription factor essential for the regulation of several pro-inflammatory enzymes such as iNOS and COX-2 [44,45]. The p65 protein of NF-κB migrates into the nucleus via LPS stimulation and facilitates the production of pro-inflammatory mediators associated with liver inflammation [46,47]. Therefore, we investigated whether LHE was effective in inhibiting MAPK/NF-κB activation in hepatic tissue lysate. In this study, Western blotting revealed that LHE was significantly effective in inhibiting the phosphorylation of all types of MAPK, including ERK, p38, and JNK, induced by LPS/D-GalN. Moreover, LHE could inhibit the activation of NF-κB p65 via the inhibition of IκBα phosphorylation. These results suggested that MAPK/NF-κB might be a pharmacological target of LHE against LPS/D-GalN-induced hepatic damage.

Nrf-2 is an important transcription factor that can induce the production of antioxidant enzymes in macrophages. Nrf-2 regulates the expression of HO-1, which is translocated into the nucleus to exert its effect. As Nrf-2 activation is also involved in the expression of iNOS and COX-2 [48], Nrf-2 plays an important role in the regulation of inflammation. Also, previous studies reported that activating of Nrf-2 exerts protective effect against LPS/D-GalN-induced liver injury [49,50]. Therefore, we investigated the effects of LHE treatment on the expression of antioxidant-related proteins. LHE treatment was shown to markedly increased expression of Nrf-2 and up-regulated the level of Nrf-2-depending signaling including antioxidant protein HO-1. LHE also significantly inhibited the expression of iNOS and COX-2 in mice liver upon LPS/D-GalN injection. These outcomes supported that LHE improved LPS/D-GalN-induced hepatic liver injury via activating the Nrf-2/HO-1 signaling pathway.

Additionally, we determined how LHE affects the inflammatory reaction in endotoxin-stimulated RAW 264.7 macrophages. LHE pretreatment at a non-toxic concentration significantly suppressed the secretion of several inflammatory mediators, such as NO, TNF-α, IL-6, and IL-1β in LPS-stimulated macrophage RAW 264.7 cells. Additionally, both protein and mRNA levels of iNOS and COX-2 were inhibited in a concentration-dependent manner by LHE. In addition, we showed that LHE dramatically increased the protein and mRNA expression of HO-1. The cytosolic protein level of Nrf-2 decreased after LHE treatment. Meanwhile, the nuclear protein level of Nrf-2 increased in a concentration-dependent manner. Thus, LHE could promote the nuclear translocation of Nrf-2, leading to HO-1 expression in RAW 264.7 cells. In addition, the activation of HO-1/Nrf-2 might be mediated by the phosphorylation of MAPK [51]. Therefore, whether LHE prevents MAPK phosphorylation and the nuclear translocation of NF-κB p65 in LPS-stimulated macrophage RAW 264.7 cells was assessed. Our data show that pretreatment of LHE remarkably inhibited the phosphorylation of MAPK and nuclear migration of NF-κB p65 in LPS-stimulated macrophages via repression of IκBα degradation. Taken together, LHE exhibits strong hepatoprotective and anti-inflammatory effects through the regulation of Nrf-2/HO-1 and MAPK/NF-κB signaling pathway.

To investigate the relationships between the physiological activities of LHE and its components, we performed phytochemical analyses using HPLC. Moreover, five main components, including protocatechuic acid, catechin, quercitrin, quercetin, and kaempferol, were identified. Previous studies have shown that protocatechuic acid has protective effects against menadione-induced liver damage by up-regulating Nrf-2 [52]. Moreover, it could suppress airway inflammation via the inhibition of MAPK [53]. In addition, quercitrin decreased the risk of acetaminophen-induced acute liver toxicity in mice [54]. Moreover, quercitrin and quercetin reduced the occurrence of inflammatory response and oxidative stress in macrophages [55]. In addition, another recent study has shown that kaempferol has mitigating effects against liver fibrosis in mice [56]. Based on the current HPLC analysis and previous studies about these components, the anti-inflammatory properties and hepatoprotective effects of LHE can likely reflect the presence of protocatechuic acid, quercitrin, quercetin, and kaempferol.

## 5. Conclusions

LHE has hepatoprotective and anti-inflammatory effects in LPS/D-GaIN-induced acute liver injury mouse model and in a LPS-stimulated macrophage RAW 264.7 cells. The mechanisms underlying these effects include the inhibition of NF-κB activation via IκBα stabilization and the reduction of MAPK phosphorylation, including ERK, P38, and JNK. Moreover, it has an antioxidant effect by enhancing HO-1/Nrf-2 activation. This, in turn, inhibits the production of inflammatory mediators, such as NO, iNOS, COX-2, and pro-inflammatory cytokines in LPS/D-GaIN-induced liver injury mouse model and the LPS-exposed macrophages. Histological evaluation showed that LHE is significantly effective against LPS/D-GaIN-induced liver injury. Moreover, some LHE components, such as protocatechuic acid, quercitrin, quercetin, and kaempferol, may be closely correlated with the hepatoprotective and anti-inflammatory effects of LHE. These in vivo outcomes indicated that LHE has anti-hepatotoxic and anti-inflammatory properties and that it has a therapeutic potential against inflammatory-related hepatic disease.

## Figures and Tables

**Table 1:** Primary and secondary antibodies use for Western blot analysis.

Antibody	Corporation	Product No.	RRID	Dilution Rate
iNOS	Cell Signaling	#13120	AB_2687529	1:1000
COX-2	Cell Signaling	#4842	AB_2085144	1:1000
HO-1	Cell Signaling	#82206	AB_2799989	1:1000
Nrf-2	Cell Signaling	#12721	AB_2715528	1:1000
P-NF-κB p65	Cell Signaling	#3033	AB_331284	1:1000
NF-κB p65	Cell Signaling	#8242	AB_10859369	1:1000
P-IκBα	Cell Signaling	#2859	AB_561111	1:1000
IκBα	Cell Signaling	#4814	AB_390781	1:1000
P-ERK	Cell Signaling	#4377	AB_331775	1:1000
ERK	Cell Signaling	#9102	AB_330744	1:1000
P-p38	Cell Signaling	#9211	AB_331641	1:1000
p38	Cell Signaling	#9212	AB_330713	1:1000
P-JNK	Cell Signaling	#9251	AB_331659	1:1000
JNK	Cell Signaling	#9252	AB_2250373	1:1000
β-actin	Cell Signaling	#4970	AB_2223172	1:1000
TBP	Cell Signaling	#8515	AB_10949159	1:1000
2nd anti-mouse	Cell Signaling	#7076	AB_330924	1:5000
2nd anti-rabbit	Cell Signaling	#7074	AB_2099233	1:5000

**Table 2:** Primers used for qPCR.

Target Gene	Reference Sequence	Primer Sequence
TNF-α	NM_013693.3	F: 5′-TTCTGTCTACTGAACTTCGGGGTGATCGGTCC-3′
		R: 5′-GTATGAGATAGCAAATCGGCTGACGGTGTGGG-3′
IL-6	NM_031168.2	F: 5′-TCCAGTTGCCTTCTTGGGAC-3′
		R: 5′-GTGTAATTAAGCCTCCGACTTG-3′
IL-1β	NM_008361.4	F: 5′-ATGGCAACTGTTCCTGAACTCAACT-3′
		R: 5′-CAGGACAGGTATAGATTCTTTCCTTT-3′
iNOS	NM_010927.4	F: 5′-GGCAGCCTGTGAGACCTTTG-3′
		R: 5′-GCATTGGAAGTGAAGCGTTTC-3′
COX-2	NM_011198.4	F: 5′-TGAGTACCGCAAACGCTTCTC-3′
		R: 5′-TGGACGAGGTTTTTCCACCAG-3′
HO-1	NM_010442.2	F: 5′-TGAAGGAGGCCACCAAGGAGG-3′
		R: 5′-AGAGGTCACCCAGGTAGCGGG-3′
β-actin	NM_007393.5	F: 5′-AGAGGGAAATCGTGCGTGAC-3′
		R: 5′-CAATAGTGATGACCTGGCCGT-3′

F, forward; R, reverse.

**Table 3:** HPLC conditions for analysis.

HPLC Conditions			
Detector	270 nm		
Column	X-bridge C_18_ (250 mm × 4.6 mm, 5 μm)		
Column Temperature	40 °C		
Injection Volume	10 μL		
Flow rate	1.0 mL/min		
Mobile phase	Time (min)	A	B
A: 1.0% Formic acid in Water B: Acetonitrile	0.0	97	3
	10.0	85	15
	50.0	50	50
	80.0	0	100

**Table 4:** Calibration curves of compounds and LOD, LOQ.

Compound	Range (μg/mL)	Regression Equation	*r* ^2^	LOD (μg/mL)	LOQ (μg/mL)
1	10.0~50.0	y = 0.4829x + 0.2831	0.9997	0.0027	0.0083
2	80.0~400.0	y = 0.0430x + 0.1581	0.9991	0.0310	0.0939
3	10.0~50.0	y = 0.3330x + 0.2098	0.9998	0.0040	0.0121
4	10.0~50.0	y = 0.4722x + 0.0151	0.9996	0.0028	0.0085
5	10.0~50.0	y =0.5990x + 0.6334	0.9977	0.0022	0.0067

Protocatechuic acid (1); catechin (2); quercitrin (3); quercetin (4); kaempferol (5). LOD = 3.3 × σ/*S*. LOQ = 10 × σ/*S*. σ is the standard deviation of the intercept from the regression equation and *S* is the slope of the calibration curve.

**Table 5:** The amount of constituents in LHE.

Compound	Content (%)
Protocatechuic acid	0.03
Catechin	0.30
Quercitrin	0.04
Quercetin	0.03
Kaempferol	0.04

## Data Availability

The data are contained within the article.
